# Acknowledgement to Reviewers of *Cancers* in 2015

**DOI:** 10.3390/cancers8010018

**Published:** 2016-01-21

**Authors:** Cancers Editorial Office

**Affiliations:** MDPI AG, Klybeckstrasse 64, CH-4057 Basel, Switzerland; cancers@mdpi.com

The editors of *Cancers* would like to express their sincere gratitude to the following reviewers for assessing manuscripts in 2015. 

We greatly appreciate the contribution of expert reviewers, which is crucial to the journal’s editorial decision-making process. Several steps have been taken in 2015 to thank and acknowledge reviewers. Good, timely reviews are rewarded with a discount off their next MDPI publication. By creating an account on the submission system, reviewers can access details of their past reviews, see the comments of other reviewers, and download a letter of acknowledgement for their records. In addition, MDPI has launched a collaboration with Publons, a website that seeks to publicly acknowledge reviewers on a per journal basis. This is all done, of course, within the constraints of reviewer confidentiality. Feedback from reviewers shows that most see their task as a voluntary and mostly unseen work in service to the scientific community. We are grateful to our reviewers for the contribution they make.

Adams-Campbell, Lucile
Agarwal, Rajesh
Agarwal, Saurabh
Alberti, Saverio
Allan, Carolyn A.
Allen, Christopher
Altomare, Donato F.
Amato, Robert J.
Angelucci, Adriano
Antoni, Delphine
Aptsiauri, Natalia
Araujo, Sofia J.
Armoogum, Kris
Augsten, Martin
Avram, Marioara
Ayad, Nagi G.
Azoury, Saïd C.
Bachmann, Andre
Bagchi, Anindya
Barbolina, Maria V.
Bastian, Boris C.
Beasley, Georgia
Becker, Donald F.
Bedogni, Barbara
Beebe, Stephen J.
Berman, Russell
Bernstein, Emily
Biasotto, M.
Biccard, B.M.
Bingham, Paul M.
Biunno, Ida
Black, Jennifer D.
Black, Morgan
Blair, Geoger Eric
Blandino, G.
Blattmann, Claudia
Bonnelykke-Behrndtz, M.L.
Bosch, Ricardo J.
Bossi, Paolo
Bouffet, Eric
Briasoulis, Evangelos
Brinkmann, Melanie
Brownell, Isaac
Burns, Erin M.
Calvisi, Diego F.
Campbell, Christopher
Campbell, Colin
Carpenter, Richard C.
Chandran, Bala
Chang, Joe Y.
Chauhan, Harsh
Chellappan, Srikumar
Chen, Xi
Chi, Sung-Gil
Chien, Andy J.
Chin, Lawrence S.
Chu, Quincy
Cicenas, Jonas
Ciocca, Mario
Clark, Geoffrey J.
Coley, Helen M.
Comincini, Sergio
Copland, Mhairi
Culig, Zoran
Deacon, Karl
Delbue, Serena
Depauw, Nick
Dhawan, Deepika
Dhermain, Frédéric Geza
Dhesi, J.K.
Di Paolo, Antonello
Dikalov, Sergey
Ding, Jian
Dinulescu, Daniela M.
Dixon, Dan
Driscoll, James J.
Eedunuri, Vijay Kumar
El-Naggar, Adel K.
Elas, Martyna
Enfield, Katey S.S.
Erovic, Boban M.
Esquela-Kerscher, Aurora
Esteve, Jordi
Evron, Ella
Falzon, Miriam
Farah, Jad Walid
Farren, Matthew
Fellowes, Andrew
Feng, Hui
Ferri, Clodoveo
Fields, Gregg B.
Filippova, Maria
Forde, Patrick
Forget, Patrice
Frampton, Adam
Franco, Omar
Funahashi, Yasuhiro
Furuno, Tadahide
Gabrielli, Brian
Ganguly, Anutosh
Gatti Luini, Maria Giovanna
Gehler, Scott R.
Gerke, Oke
Ghabrial, Amin
Giandomenico, Valeria
Giantsoudi, Drosoula
Giardina, Charles
Giraudo, Claudio G.
Girbes, T.
Gisselbrecht, Christian
Gorgoulis, Vassilis G.
Gouilleux-Gruart, Valerie
Gram, Inger Torhild
Green, Victoria
Gridley, Daila S.
Grippo, Paul J.
Gruchy, N.
Gruvberger-Saal, Sofia K.
Guerrero, Carmen
Guo, Z. Sheng
Guo, Zhenheng
Haick, Hossam
Hamdy Roby, M.H.
Hartmann-Johnsen, O.J.
Hatt, Mathieu
Haustermans, Karin
Hedegaard, Jakob
Held, Kathy
Heller, Richard
Hermann, Patrick Christian
Hernandez-Ilizaliturri, F.
Hesson, Luke
Hinds, Philip W.
Hishikawa, Yoshio
Hoeller, Christoph
Hohaus, Stefan
Honeychurch, Jamie
Hoppe, Bradford S.
Horsman, Michael R.
Hortobagyi, Tibor
Hsieh, Chung-Bao
Hua, Chia-ho
Huang, Rwei-fen S.
Huang, Jianguo
Huber, Peter
Hui, Chi-chung
Ibraheem, I.
Inaguma, Shingo
Ishida, Mitsuaki
Ishitsuka, Kenji
Itano, Naoki
Malemud, Charles
Jackisch, Christian
Jalili, Ahmad
Jan, Lily Yeh
Jazirehi, Ali
Jenkins, Brenda Campbell
Jiao, Li
Jonckheere, Nicolas
Joseph, Richard
Jueckstock, J.
Juzeniene, Asta
Kammula, Udai S.
Kapustin, Alexander
Karkhanis, Vrajesh
Kato, Takamitsu A.
Khare, Sharad
Kieber-Emmons, Thomas
Kim, Yon Hui
Kim, Sang-We
Kim, Seung Jin
Klonisch, Thomas
Knecht, Hans
Kong, Feng-Ming
Konnick, Eric
Korc, Murray
Korlach, Jonas
Kruit, Wim H.J.
Kuno, Hirofumi
Kuo, Ming-Liang
Kuo, Sung-Hsin
Lace, Michael J.
Lambert, Daniel W.
Landolfo, Santo
LaRue, Amanda C.
Lathia, Justin
Lauth, Matthias
Law, Matthew H.
Lee, Mon-Juan
Lee, J.-H.
Lee, Tzong-shyuan
Lee, Bong Hwa
Lengyl, Ernst
Leporatti, Stefano
Leung, G.K.K.
Li, Zuofeng
Li, Yi
Li, Shengwen Calvin
Li, Bing
Li, Wen
Li, Hua
Lin, Haibo
Lin, Ruiting
Linz, Ute
Liszka, Łukasz
Litton, Jennifer
Liu, Gang
Liu, Qi
Liu, Zhiqiang
Liu, Jun O.
Liu, Yunhua
Louie, Stan
Lowery, Jan T.
Ludwig, Nicole
Ma, Shuang
Maggiolini, Marcello
Magnusson, Lisa U.
Mak, King Lun Kingston
Manolopoulos, Spyros
Mao, Junhao
Marini, Pietro
Martens, John Wm
Martín-Cordero, Carmen
Martinelli, Giovanni
Martinez, Jesse D.
Martinez, Inigo
Maser, Raelene E.
Mazzoccoli, Gianluigi
McCalmont, Timothy H.
McCann, Amanda
McCullagh, Laura
McLaren, Christine E.
McLaughlin-Drubin, M.
Merz, Felicitas
Meyer, Juergen
Meyer, Jeffrey
Micali, Giuseppe
Michael, Michael
Michiels, Carine
Mitsunaga, Shuichi
Miyazawa, Keiji
Moens, Ugo
Morgan, Iain
Mosialos, George
Müller-Newen, Gerhard
Murphy, Colin T.
Mus-Veteau, Isabelle
Naccarati, Alessio
Nakao, Atsuhito A.
Neumann, Joachim
Nielsen, Finn C.
Nishimura, Noriyuki
Nogueira, Veronique
Numico, Gianmauro
Ohba, Shinsuke
Okayasu , Ryuichi
Okazaki, Isao
Olofsson, Roger
Orucevic, Amila
Oshima, Tadayuki
Otey, Carol
Ow, Thomas
Ozawa, Masayuki
Padmanabhan, Jaya
Pan, Yan
Papagerakis, Silvana
Pardo, Luis
Pardo, Olivier E.
Patel, Sapna P.
Peled, Nir
Peppelenbosch, Maikel
Pérez-Andújar, Angelica
Pérol, Maurice
Perry, George
Petoukhova, A.L.
Piché, A.
Pilkington, Geoffrey
Pouliot, Normand
Powell, Joann
Prevarskaya, Natalia
Prieto, Victor G.
Prince, M.E.
Puzanov, Igor
Ramos, Juan Carlos
Ren, Jinqi Jack
Resh, Marilyn D.
Reyes, José C.
Rice, Lori
Rinnerthaler, Gabriel
Riobo, Natalia A.
Rizos, Helen
Robbins, David J.
Roditis, Maria L.
Rodriguez, Cristina P.
Rubin, Joshua B.
Rushworth, Stuart
Russo, Patrizia
Rutenberg, Michael
Rutkowski, T.
Safe, Stephen
Said, Hm
Salo, Tuula
Sand, Michael
Sanders, Niek
Sartini, Davide
Sato, Takami
Savaskan, Nicolai E.
Saw, Robyn P.M.
Schaible, Bettina
Schneider, Sallie Smith
Schweizer, Michael
Searle, Peter F.
Seelaender, Marilia
Setaluri, Vijayasaradhi
Sethi, Neeraj
Seto, Masao
Setoguchi, Takao
Shackelford, David B.
Shah, Supriya
Shah, Nilay
Sharma, Amrish
Sharma, Neelesh
Shevde, Lalita
Shi, Jiaqi
Shidong, Jia
Shiozawa, Yusuke
Sibilia, Maria
Silecchia, Gianfranco
Simiele, Felice
Simmen, Frank
Simmons, Rebecca G.
Singh, Rajendra K.
Singh, Prashant
Singh, R.R.
Slack, Frank
Slominski, Andrzej
Smith, David I.
Soloff, Adam
Spiotto, Michael T.
Stalpers, L.J.A.
Stander, Sonja
Stead, Lucy F.
Stepanova, Victoria
Sterling, Julie Ann
Strigari, Lidia
Studzinski, George P.
Sun, Beicheng
Sun, Zhonghua
Suzuki, Yuichiro J.
Tabocchini, Maria Antonella
Takano, Shinichi
Takigawa, Nagio
Tana, Claudio
Taskén, Kristin Austlid
Terracciano, Luigi
Teti, Diana
Thomas, Sufi Mary
Thrower, Edwin
Thunnissen, Erik
Thyagarajan, Bharat
Tian, Zhen
Tien, Hwei-Fang
Tikkanen, Ritva
Tinhofer-Keilholz, Ingeborg
Tomiyasu, Hirotaka
Toth, Zsolt
Treaba, Diana O.
Trevino, Jose
Tugues Solsona, Sonia
Turnbull, Jeremy
Umemura, Shigeki
van Ballegooijen, Hanne
Van Den Brekel, Michiel
Van Leeuwen, Barbara L.
Vasavada, Rupangi C.
Vidi, Pierre
Villagra, A.
Viola, Giampietro
Volante, Marco
Wada, Ryuichi
Wahlestedt, Claes
Wakatsuki, Masaru
Wakimoto, Hiroaki
Walterhouse, David O.
Wan, Liling
Wang, Kepeng
Wang, Shao-Chun
Wang, Ying
Watanabe, Jun
Weed, Donald
Weeraratna, Ashani T.
Weigert, Oliver
Weissman, Allan
Wieser, Rotraud
Winquist, Raymond
Wittel, Uwe
Wong, Chun-Ming
Woo, Shiao
Wu, Xin
Wu, Erxi
Xie, Guofeng
Xie, Jingwu
Xie, Hong
Xu, Xiulong
Yamada, Tesshi
Yang, Gary Y.
Yang, Jer-Yen
Yang, Xiuwei H.
Yang, Fei
YAP, Celestial T.
Yarnold, John
Yeddula, Narayana
Yeung, Daniel
You, Yongping
Yu, Cheng-Chia
Yu, Victor
Yun, Yang H.
Zage, Peter E.
Zakharian, Eleonora
Zanetti, Maurizio
Zaphiropoulos, Peter
Zhang, Hao
Zhang, Caiguo
Zhao, Li
Zhao, Jianjun
Zheng, Shu-sen
Zielinski, Christoph C.

